# Non-invasive detection of 2-hydroxyglutarate in IDH-mutated gliomas using two-dimensional localized correlation spectroscopy (2D L-COSY) at 7 Tesla

**DOI:** 10.1186/s12967-016-1035-1

**Published:** 2016-09-22

**Authors:** Gaurav Verma, Suyash Mohan, MacLean P. Nasrallah, Steven Brem, John Y. K. Lee, Sanjeev Chawla, Sumei Wang, Rajakumar Nagarajan, M. Albert Thomas, Harish Poptani

**Affiliations:** 1 Division of Neuroradiology, Department of Radiology, Perelman School of Medicine at University of Pennsylvania, 3400, Spruce St., Philadelphia, PA 19014 USA; 2 Department of Pathology, University of Pennsylvania, Philadelphia, PA USA; 3 Department of Neurosurgery, University of Pennsylvania, Philadelphia, PA USA; 4 Department of Radiological Sciences, UCLA, Los Angeles, CA USA; 5 Department of Cellular and Molecular Biology, University of Liverpool, Liverpool, UK

**Keywords:** 2-Hydroxyglutarate, Isocitrate dehydrogenase, Phosphocholine, Correlation spectroscopy, Brain tumor

## Abstract

**Background:**

Mutations in the isocitrate dehydrogenase enzyme are present in a majority of lower-grade gliomas and secondary glioblastomas. This mis-sense mutation results in the neomorphic reduction of isocitrate dehydrogenase resulting in an accumulation of the “oncometabolite” 2-hydroxyglutarate (2HG). Detection of 2HG can thus serve as a surrogate biomarker for these mutations, with significant translational implications including improved prognostication. Two dimensional localized correlated spectroscopy (2D L-COSY) at 7T is a highly-sensitive non-invasive technique for assessing brain metabolism. This study aims to assess tumor metabolism using 2D L-COSY at 7T for the detection of 2HG in IDH-mutant gliomas.

**Methods:**

Nine treatment-naïve patients with suspected intracranial neoplasms were scanned at 7T MRI/MRS scanner using the 2D L-COSY technique. 2D-spectral processing and analyses were performed using a MATLAB-based reconstruction algorithm. Cross and diagonal peak volumes were quantified in the 2D L-COSY spectra and normalized with respect to the creatine peak at 3.0 ppm and quantified data were compared with previously-published data from six normal subjects. Detection of 2HG was validated using findings from immunohistochemical (IHC) staining in patients who subsequently underwent surgical resection.

**Results:**

2HG was detected in both of the IDH-mutated gliomas (grade III Anaplastic Astrocytoma and grade II Diffuse Astrocytoma) and was absent in IDH wild-type gliomas and in a patient with breast cancer metastases. 2D L-COSY was also able to resolve complex and overlapping resonances including phosphocholine (PC) from glycerophosphocholine (GPC), lactate (Lac) from lipids and glutamate (Glu) from glutamine (Gln).

**Conclusions:**

This study demonstrates the ability of 2D L-COSY to unambiguously detect 2HG in addition to other neuro metabolites. These findings may aid in establishing 2HG as a biomarker of malignant progression as well as for disease monitoring in IDH-mutated gliomas.

## Background

Mutations in the gene encoding isocitrate dehydrogenase (IDH) occur in up to 80 % of World Health Organization (WHO) grade II/III gliomas and a majority of secondary glioblastomas (GBMs) [1–3]. Glioma patients, whose lesions harbor IDH mutations are associated with better prognosis, are more sensitive to chemo-radiation therapy and demonstrate longer survival than those with wild-type IDH [4, 5]. These mis-sense mutations may confer the neomorphic ability to reduce isocitrate dehydrogenase, resulting in the accumulation of the “oncometabolite” 2-hydroxyglutarate (2HG) [6, 7] rather than its normal catalysis into α-ketoglutarate. IDH-mutation status has taken on greater clinical significance in this era of molecular diagnostics, with recent major restructuring of central nervous system (CNS) tumors as published in the 2016 WHO classification [8].

In addition to 2HG, several other metabolic processes are altered in brain tumors. A prominent resonance from total choline comprising of free choline (Cho), phosphocholine (PC) and glycerophosphocholine (GPC) is commonly observed in conventional 1D proton magnetic resonance spectroscopy (^1^H MRS) studies of brain tumors. However, in order to properly understand the dysregulated Cho metabolism, it is important to detect and quantify relative levels of PC, a vital indicator for the expression and/or activity of choline kinase [9] and GPC. Also important is to understand the alteration in the glutamate (Glu), glutamine (Gln) and gamma-aminobutyric acid (GABA) cycle as Glu is involved in the cellular anabolic pathways and in facilitating tumor invasion [10].

Non-invasive detection of 2HG and other resonances on conventional ^1^H MRS is challenging due to extensive overlap with the resonances of neighboring metabolites. Previous ^1^H MRS studies have employed two different acquisition and processing strategies to resolve 2HG from overlapping resonances in vivo. Choi et al. [11, 12]. used a modified chemical-shift imaging (CSI) sequence with empirically-optimized pulse timing and a prior-knowledge based fitting to detect 2HG [11]. This sequence employs an echo time (TE) of 97 ms to detect resonances correspond to 2HG, but this comes with the limitation that quantification of other metabolites then becomes more difficult. A second strategy to detect 2HG is to use two-dimensional localized correlated spectroscopy (2D L-COSY) to introduce a second spectral dimension and identify 2HG by “cross-peak” resonances due to J-coupling interactions [13]. Using in vivo 2D L-COSY [14, 15], Ramadan et al. [16] have reported the detection of several metabolites such as PC, GPC, Lac and Glu/Gln from GBMs, indicating a potential utility of 2D L-COSY in further characterization of brain neoplasms. Though well-separated, these cross-peaks show lower signal intensity than primary resonances because they originate only from the small fraction of nuclei which undergo coherence transfer during t_1_-evolution, motivating a move to higher field strengths.

The introduction of ultrahigh field (7T) scanners improves the sensitivity of ^1^H MRS in the detection of 2HG and other complex metabolites due to greater signal to noise ratio and increased chemical shift dispersion. We have recently implemented 2D L-COSY at 7T [17], to reliably identify metabolites that were otherwise difficult to resolve with conventional ^1^H MRS, especially at lower field strength [11]. In this study, we aimed to test the efficacy of 2D L-COSY at 7T for detection of 2HG in IDH-mutated gliomas.

## Methods

### Subjects

The study was approved by the Institutional Review Board of the University of Pennsylvania and is compliant with the Health Insurance Portability and Accountability Act. Written informed consent was obtained from all patients prior to the study. A total of nine patients (five female, four male, ages 18–72 years, mean = 42 years) with suspected intracranial neoplasms based on clinical presentation and routine 3T MR imaging findings were recruited for this study. All 7T scans were performed following initial diagnosis and prior to surgical biopsy/tumor resection and chemo-radiation therapy. Histopathological and immunohistochemical analyses on the resected tumor specimens were subsequently performed and the findings were compared with the results from spectral data, specifically for tumor grade and IDH mutation status.

### MR imaging and spectroscopy

All scans were performed on a Siemens (Siemens Healthcare, Erlangen, Germany) 7T MRI/MRS whole-body scanner equipped with a 32-channel transmit/receive head coil. Axial T_2_-weighted fluid-attenuated inversion recovery (FLAIR) imaging was used to facilitate voxel localization on the intracranial neoplasms. Imaging parameters for the FLAIR sequence included: Echo time (TE) = 382 ms, repetition time (TR) = 4500 ms, TI = 2150 ms, 1 average, 240 × 240 mm^2^ field of view (FOV), 320x320 matrix size, 208 slices, 0.8 × 0.8 × 0.8 mm^3^ resolution, 6 min scan time.

A single voxel 2D L-COSY sequence was performed by selecting a voxel size of 8.8–18.0 ml from the solid portion of the neoplasm, carefully avoiding regions of hemorrhage/cysts. 2D L-COSY scan parameters were as follows: TE = 20 ms, TR = 2000 ms, 8 averages for each incremental period, 64 ∆t_1_ increments of 0.4 ms, 2048 t_2_ points with F_2_/F_1_ bandwidth = 4000/2500 Hz with a total scan time of 17 min. B_1_ field optimization was assisted by the automated FASTESTMAP (Siemens Healthcare) algorithm and water suppression was performed using the variable power and optimized relaxation delays (VAPOR) module [18].

Spectra from neoplasms were compared with data obtained from six healthy controls as part of a previous study [19]. In that study a voxel size of 27.0 ml encompassing bilateral cortical occipital region was used in all subjects. All other scan parameters for the control subjects were identical to those for the patients.

### Data processing

Data from the 2D L-COSY studies were reconstructed offline using a custom MATLAB-based reconstruction program. Post-processing steps included Fourier transform, zero-filling, apodization using a squared shifted sine-bell windowing function and combining coil and averaging data. Metabolite signals were quantified through peak volume integration. The diagonal resonance of creatine (Cr) at [F_2_, F_1_] = 3.0, 3.0 ppm was used as an internal reference for computing metabolite ratios. Chemical shifts were calibrated using the resonance of NAA at [F_2_, F_1_] = 2.0, 2.0 ppm. Detection of 2HG was used to prospectively determine IDH mutation status and these findings were subsequently compared with the results from pathology.

### Determination of IDH Status by Immunohistochemistry

Patient demographics along with histopathological grading and immuno-histochemical findings are presented in Table 1. Of the nine patients, one participant (patient #3) expressed physical discomfort during the scan and requested to terminate the study prematurely. 2D L-COSY data from two other subjects (patients #7 and #8, Table 1) had sub-optimal water suppression and produced spectra of insufficient quality for reliable metabolite quantification. Both of these spectra featured a broad resonance due to the residual signal from unsuppressed water in the region where the resonances due to 2HG, GPC, PC and Lac would be expected.

**Table 1:** Patient demographics and histological diagnosis

ID	Age (years)	Sex	Histopathology results	WHO grade	IDH1 status	Spectral quality
1	45	F	Hematoma		Not tested	Good
2	49	F	Metastatic breast primary		Not tested	Good
3	32	F	Low grade glioma (clinical MRI)		Not available	N/A
4	58	F	Ganglioglioma	I	Wild-type	Good
5	43	F	Ganglioglioma/xanthoastrocytoma	I/II	Wild-type	Good
6	18	M	Diffuse astrocytoma	II	Mutant	Good
7	22	M	Astrocytoma	II	Mutant	Poor
8	72	M	Anaplastic astrocytoma	III	Wild-type	Poor
9	36	M	Anaplastic astrocytoma	III	Mutant	Good

Tissue specimens received by pathology were fixed in formalin and processed for paraffin embedding. Hematoxylin and eosin staining and immunohistochemistry were conducted on 5-micron thick formalin-fixed, paraffin-embedded tissue sections mounted on Leica Surgipath slides followed by drying for 60 min at 70°. Immunohistochemistry with the anti-IDH1-R132H antibody (Monoclonal Mouse Anti-human IDH1 (R132H), Dianova, DIA Clone H09) and DAB chromogen was performed on a Leica Bond III instrument using Bond Polymer Refine Detection System (Leica Microsystems AR9800) following a 20-min heat-induced epitope retrieval with Epitope Retrieval 2, EDTA, pH 9.0.

## Results

**Fig. 1:**
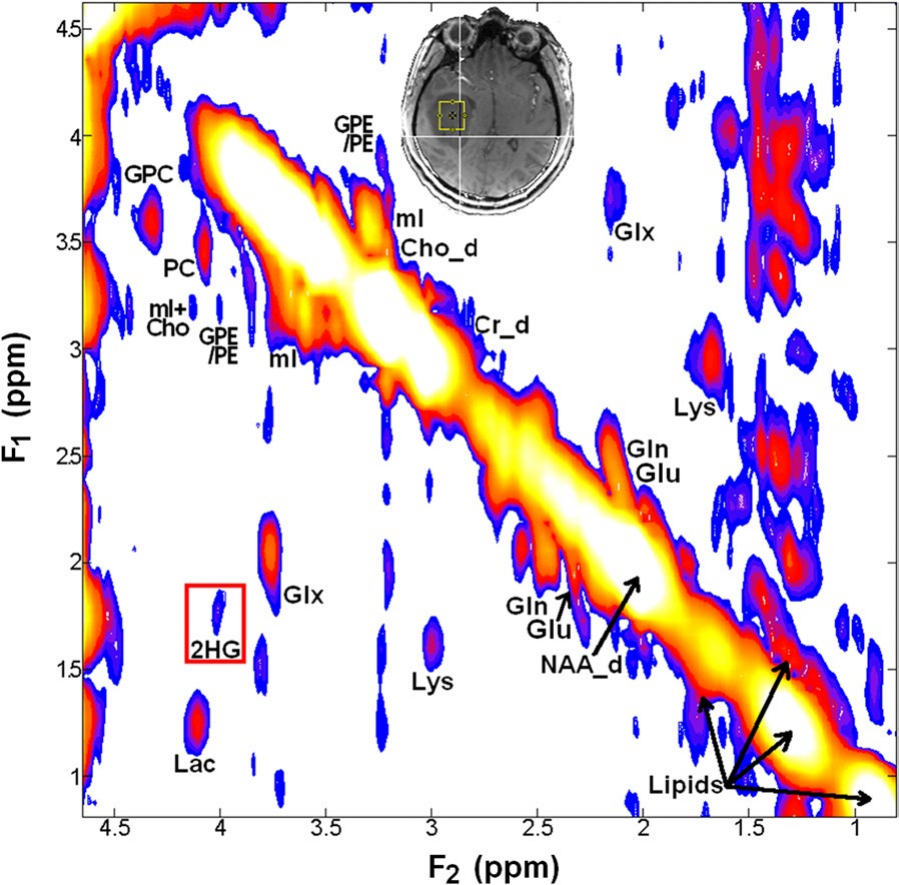
7T COSY spectrum from an 11.0 ml voxel localized within a patient with mutant-IDH1 WHO grade III anaplastic astrocytoma (*middle inset*). Metabolite peaks including the resonance peak due to 2HG have been labeled. The cross peaks from 2HG at [F_2_, F_1_] = 4.0, 1.7 ppm are identified within the *red box*

**Fig. 2:**
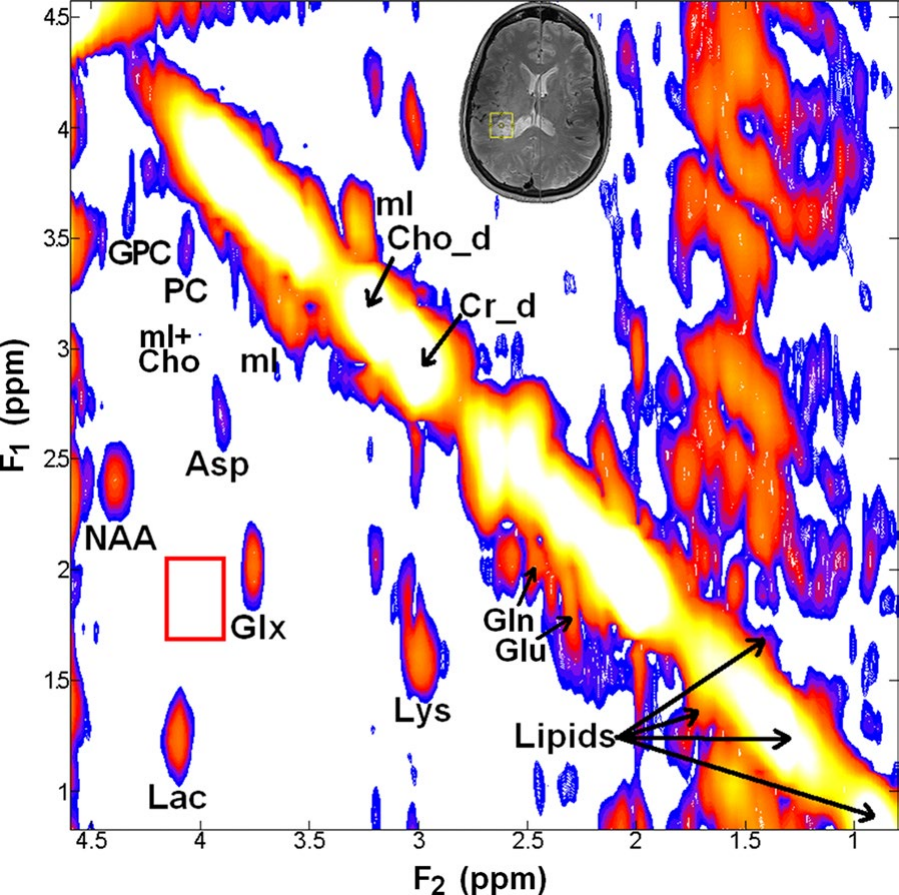
7T COSY spectrum from an 8.8 ml voxel localized in a patient with wild-type IDH1 WHO grade 1 ganglioglioma (*middle inset*) showing absence of cross peaks from 2HG (*empty red box* around [F_2_, F_1_] = 4.0, 1.7 ppm)

2D L-COSY spectra showed presence of 2HG at [F_2_, F_1_] = 4.0, 1.7 ppm in two of the six glioma cases (grade III anaplastic astrocytoma and grade II diffuse astrocytoma). None of wild-type IDH1 patients demonstrated the 2HG peak on the 2D L-COSY spectrum. IHC confirmed IDH1 mutation in both patients whose spectra showed presence of 2HG and IDH1 mutation was not detected in any of the other glial tumors studied. 2D L-COSY spectrum from a tumor harboring IDH1 mutation (patient #9) is shown in Fig. 1 with the voxel localization for the MRS shown on the T_2_-FLAIR image (inset). The diagonal peaks and cross-peak resonances due to coherence transfer in J-coupled metabolites are well-resolved and some of them have been labeled as: 2HG, Lac, lipids, GPC, PC, GPE + PE and mI + Cho. Representative 2D L-COSY spectrum from a patient with a wild-type IDH1 (patient #4) is shown in Fig. 2.

Besides NAA, Cr, tCho and mI that are typically resolved on 1D ^1^H MRS of brain neoplasms, resonances corresponding to other metabolites, e.g. PC, GPC, Glu, Gln, Lac and lipids were clearly resolved on 2D L-COSY spectra in each of the six patients. In addition, we also observed resonances corresponding amino acids such as aspartate (Asp) and lysine (Lys) not generally detected on 1D ^1^H MRS (Figs. 1, 2).

**Fig. 3:**
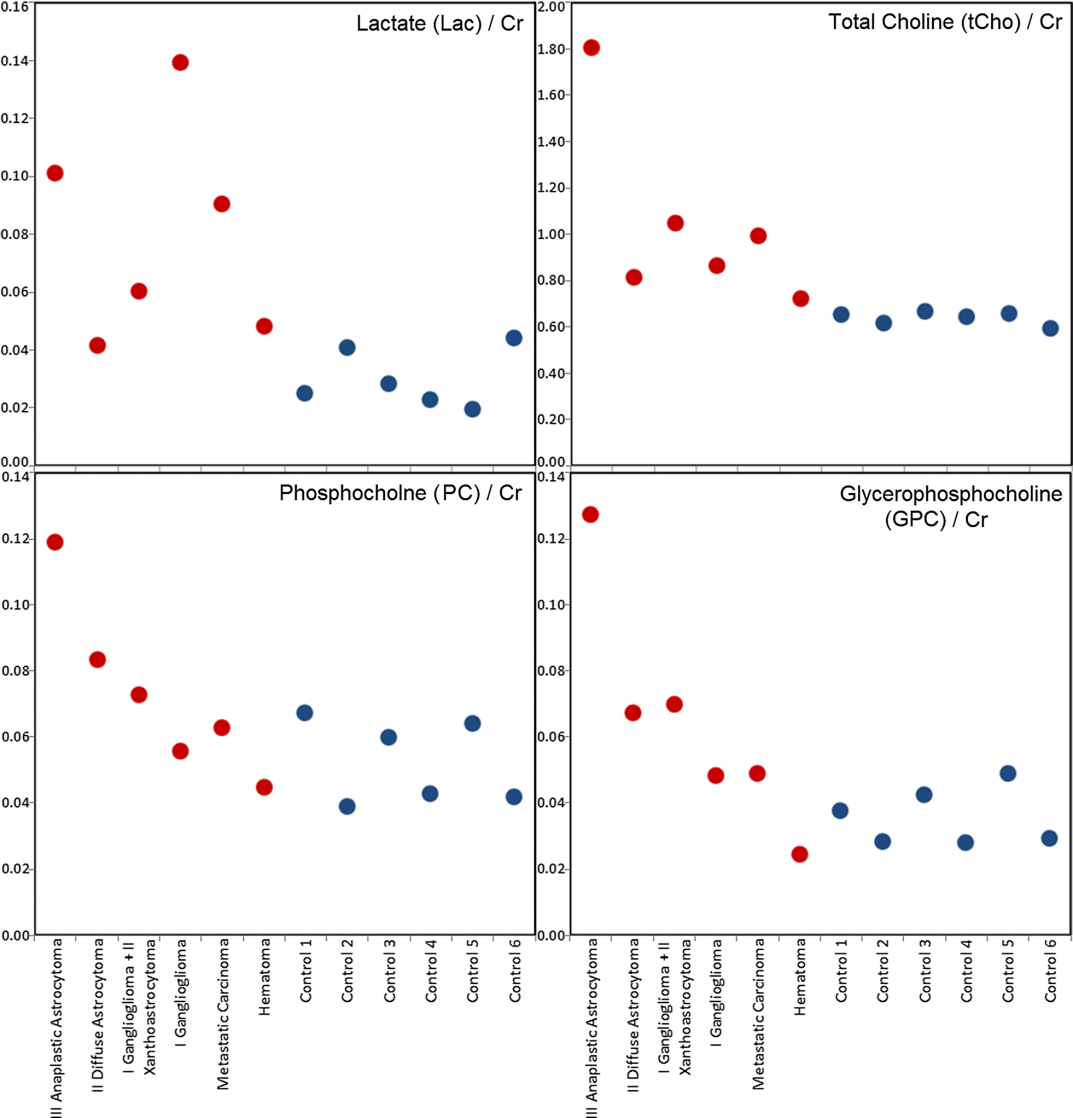
Metabolite/Cr ratios for Lac, Cho, PC and GPC measured in L-COSY data from each of the six patients (*red*) and six controls (*blue*)

Figure 3 shows ratios of Lac and tCho with respect to creatine in each of the six brain tumor patients and normal controls. GPC/Cr and PC/Cr ratios were high in the only WHO grade III tumor studied, while these ratios were indistinguishable from normal controls in the patient with hematoma. An increasing trend in GPC, PC and total Cho was noticed with increasing tumor grade. No such trend was observed for the Lac signal.

Table 2 shows a list of ratios of cross-peak metabolite resonances with respect to Cr from the six patients and six normal subjects. These metabolites included Lac, lipids, lysine (Lys), Glu, Gln (and their combined resonance, Glx), NAA, Asp, mI, glycerophosphoethanolamine (GPE) and phosphoethanolamine (PE), the combined cross peaks of mI and free choline (mI + Cho), GPC and PC.

**Table 2:** Metabolite ratios with respect to Cr in patients and normal controls from the L-COSY data

Metabolites (Cr)	Chemical shift		Patient 1	Patient 2	Patient 4	Patient 5	Patient 6	Patient 9	Controls (N = 6)
	F_2_ (ppm)	F_1_ (ppm)	N/A	N/A	Grade I	Grade I/II	Grade II	Grade III	N/A
NAA-d	2.0	2.0	1.80	1.29	1.49	1.06	1.29	1.21	1.37
Cr-d	3.0	3.0	1.00	1.00	1.00	1.00	1.00	1.00	1.00
Cho-d	3.2	3.2	0.72	0.99	0.86	1.05	0.81	1.81	0.64
mI-d	3.5	3.5	0.36	0.38	0.38	0.68	0.45	0.82	0.28
Lipid-d	0.9	1.3	0.78	1.67	1.09	0.91	0.59	1.25	ND
Lac	4.1	1.3	0.05	0.09	0.14	0.06	0.04	0.10	0.01
Lys	3.0	1.7	0.14	0.12	0.16	0.11	0.12	0.11	0.10
GABA	3.0	1.9	0.10	0.08	0.15	0.11	0.08	0.08	0.07
Glu	2.4	2.0	0.21	0.23	0.21	0.20	0.24	0.19	0.23
Gln	2.5	2.1	0.19	0.39	0.19	0.19	0.21	0.26	0.20
Glx	3.7	2.1	0.09	0.15	0.09	0.11	0.11	0.14	0.17
NAA	4.4	2.5	0.05	0.03	0.04	0.02	0.04	0.03	0.05
Asp	3.9	2.8	0.03	0.04	0.05	0.07	0.05	0.07	0.05
Tau	3.4	3.2	0.14	0.12	0.16	0.12	0.08	0.19	0.09
mI	3.6	3.3	0.23	0.24	0.31	0.30	0.30	0.53	0.24
GPE/PE	3.9	3.2	0.04	0.06	0.04	0.06	0.13	0.11	0.04
2HG	4.0	1.7	ND	ND	ND	ND	0.08	0.10	ND
mI + Cho	4.1	3.3	0.04	0.06	0.04	0.05	0.07	0.08	0.06
PC	4.2	3.6	0.04	0.06	0.06	0.07	0.08	0.12	0.07
GPC	4.3	3.7	0.02	0.05	0.05	0.07	0.07	0.13	0.04
GSH	4.5	2.9	0.03	0.04	0.04	0.07	0.04	0.05	0.03

The first five rows represent data from resonances that appear as diagonal peaks (indicated by -d)

*ND* not detected

## Discussion

To our knowledge, this is the first study of its kind in which 2D L-COSY has been used to characterize brain tumors at ultrahigh fields. The proportionately higher spectral separation on the 7T scanner improved specificity of detection of 2HG as well as other highly relevant metabolites in tumors, such as GPC, PC, Lac, Lys, Glu and Gln that are difficult to unambiguously resolve at lower fields.

Previous studies have reported that over 50 % of WHO Grade II/III gliomas harbor IDH mutations [1, 2]. In fact, the 2016 update to the WHO Classification of Tumors of the CNS has made this molecular change part of the diagnosis for the grade II/III “diffuse astrocytoma, IDH-mutant,” “anaplastic astrocytoma, IDH-mutant,” “oligodendroglioma, IDH-mutant and 1p/19q-codeleted” and “anaplastic oligodendroglioma, IDH-mutant and 1p/19q-codeleted.” Grade II “diffuse astrocytoma, IDH-wildtype” is a provisional entity given its rarity [8]. 2HG has been considered as a putative biomarker of these genotypes. A previous study [20] reported that patients with gliomas bearing IDH mutations show better response to chemo-radiation therapy and thus present favorable clinical outcome corroborating the need for 2HG detection to guide clinical management [20–23]. These mutations may also be candidates for targeted therapy (e.g. AGIOS 121) [24], making reliable detection of IDH mutation increasingly relevant to both the selection and monitoring of treatment efficacy, providing real-time feedback as a robust biomarker. Although IDH mutations can be identified by immunohistochemical analysis of resected tumor specimens [25–27], non-invasive detection of 2HG levels using ^1^H MRS techniques can be used to monitor targeted therapy. Detection of these biomarkers with 7T 2D L-COSY may inform the decision for change in therapy.

We observed 2HG in each of the two patients with gliomas bearing IDH mutation and in none of the four wild-type tumors, thus substantiating the usefulness of 7T 2D L-COSY in resolving the complex signals of 2HG overlapping with other metabolites. 1D ^1^H MRS studies have recently demonstrated [5, 11, 13, 28–31] in vivo detection of 2HG in patients with gliomas harboring IDH mutation. These published studies have typically been performed on clinical 3T scanners and relied on modified sequences with optimized echo-timing to resolve complex resonances of 2HG from Glu, Gln and GABA. This may limit the signal quality from other metabolites and thus confound their reliable quantification. Both of the 7T 1D MRS reporting 2HG utilized relatively long TEs (78 and 110 ms) for the purposes of signal optimization [12, 31], which may again limit assessment of short-T_2_ metabolites like Glu and Gln, particularly at such high field. It appears that 2D L-COSY represents an alternative technique for unambiguously detecting 2HG while simultaneously identifying additional clinically-relevant metabolites with high sensitivity.

In tumors, elevated total Cho levels, detected by ^1^H MRS, is indicative of increased cell membrane turnover and tumor malignancy [32, 33]. Moreover, relative amounts of PC and GPC have been suggestive of markers for predicting tumor grade [34] using in vitro ^1^H MRS. In an in vitro ^1^H MRS study of tumor extracts [34], it was reported that PC was the predominant total choline (tCho) peak in high grade gliomas, while GPC dominates in low grade gliomas and normal brain. The higher PC in high grade gliomas has been attributed to higher expression and/or activity of choline kinase, phospholipase C and Cho transporters that have been exploited as potential targets for therapy [35]. Furthermore, alterations in the PC/GPC ratio have been proposed as a marker of malignant transformation and treatment response [36]. Taken together, these studies provide adequate impetus to separate PC from GPC in vivo, to study tumor phospholipid metabolism, which can be studied using the 2D L-COSY technique as demonstrated in this study.

Detection of elevated Lac in neoplasms in our study is consistent with previous studies [37–39] and may reflect elevated tumor glycolysis and/or poor tissue perfusion. However, reliable detection of Lac on 1D ^1^H MRS is problematic due to presence of intense co-resonant lipids signals that are also elevated in brain neoplasms [40] and may also be present as an artifact from skull, as seen in patient #4 (Fig. 2). 2D L-COSY facilitates separation of Lac from background lipid signals through detection of the Lac cross-peak.

Brain neoplasms exploit Glx transporter expression and function to alter Glu-Gln homeostasis, which supports their growth, invasion, and survival [41]. Closely coupled to Glx, the role of GABA has also been studied in brain neoplasms [42, 43]. Consequently, reliable detection of these metabolites is valuable for studying tumor metabolism. In the present study, cross-peaks of Glu, Gln and GABA were clearly observed which may aid in better understanding of the tumor metabolism.

Spectral acquisition methods such as multiple quantum filtered spectroscopy and spectral editing techniques have been used to unravel potentially overlapping resonances [44]. However, technical restrictions associated with these sequences such as sensitivity to motion artifacts and constraint of observing only a single metabolite at a time render these techniques less attractive to study brain neoplasms in vivo [44]. On the other hand, 2D L-COSY provides improved signal, better dispersion of J-coupled peaks and detection of multiple metabolites in a single recording [15]. 2D L-COSY at 7T unambiguously resolved many overlapping resonances with improved signal and better chemical shift dispersion compared to 1D MRS or studies at lower field strengths [19] suggesting the increased sensitivity of ultra-high field 2D L-COSY in studying brain neoplasms as shown in this study.

This study was limited in sample size and by heterogeneity of tumor types primarily because the purpose was simply to demonstrate the feasibility of the sequence in assessing brain tumor metabolism and detecting 2HG. Follow-up studies are needed to focus on metabolic differences in specific tumor types. We acknowledge the limitations of our 2D-LCOSY technique with respect to 1D MRS in terms of relatively longer acquisition time. Future modifications of this sequence would benefit from acceleration techniques like matched accumulation [45] or sparse sampling to reduce overall scan time. Metabolite quantification of resonances detected in 2D L-COSY could also be improved by implementing a prior-knowledge based fitting approach analogous to LCModel [46] fitting on 1D MRS rather than the peak integration method that was used in the present study. ProFit [47] is one such 2D prior-knowledge based fitting algorithm, and adopting this program to 7T studies could improve the metabolite quantification.

## Conclusion

We have demonstrated that 2D L-COSY has the ability to detect 2HG in vivo which has significant translational implications. We believe that 2D L-COSY may further improve our understanding of tumor physiology and metabolism and thus assist in better characterizing brain tumor genotypes in an era of molecular diagnostics and personalized medicine. As targeted therapies are developed for the IDH pathway, 2D-L-COSY could be used to detect 2HG as a key biomarker to evaluate treatment efficacy in a dynamic, iterative, real-time fashion, optimizing clinical trials and patient outcomes.
